# Turning Observed Founder Alleles into Expected Relationships in an Intercross Population

**DOI:** 10.1534/g3.118.200752

**Published:** 2019-02-04

**Authors:** Jilun Meng, Manfred Mayer, Erika Wytrwat, Martina Langhammer, Norbert Reinsch

**Affiliations:** Institute of Genetics and Biometry, Leibniz Institute for Farm Animal Biology (FBN), 18196 Dummerstorf, Germany

**Keywords:** founder genomic relationships, X-chromosomal genomic relationships, sex-linked inheritance, litter size, growth traits, selection experiments

## Abstract

Pedigree-derived relationships for individuals from an intercross of several lines cannot easily account for the segregation variance that is mainly caused by loci with alternative alleles fixed in different lines. However, when all founders are genotyped for a large number of markers, such relationships can be derived for descendants as expected genomic relationships conditional on the observed founder allele frequencies. A tabular method was derived in detail for autosomes and the X-chromosome. As a case study, we analyzed litter size and body weights at three different ages in an advanced mouse intercross (29 generations, total pedigree size 19,266) between a line selected for high litter size (FL1) and a highly inbred control line (DUKsi). Approximately 60% of the total genetic variance was due to segregation variance. Estimated heritability values were 0.20 (0.03), 0.34 (0.04), 0.23 (0.03), 0.41 (0.03) and 0.47 (0.02) for litter size, litter weight and body weight at ages of 21, 42 and 63 days, respectively (standard errors in brackets). These values were between 12% and 65% higher than observed in analyses that treated founders as unrelated. Fields of applications include experimental populations (selection experiments or advanced intercross lines) with a limited number of founders, which can be genotyped at a reasonable cost. In principle any number of founder lines can be treated. Additional genotypes from individuals in later generations can be combined into a joint relationship matrix by capitalizing on previously published approaches.

The founders of a pedigreed population are the very first individuals with no further recorded ancestors. They are usually treated as unrelated and non-inbred for setting up relationship matrices. However, treating founders of a genealogy as related has been shown to be a useful concept (Legarra *et al.* 2015) when genomic relationships (VanRaden 2008) and pedigree information are to be combined into a joint relationship matrix (Legarra *et al.* 2009; Aguilar *et al.* 2010). This has led to the notion that identity by descent (IBD) of founder alleles arises with a certain probability as a consequence of a limited effective population size. The main achievement of taking founder relatedness into account is a suitable scaling of pedigree relationships (Legarra *et al.* 2015), which makes them compatible with genomic relationships. Other benefits are reasonably interpretable estimates of genetic variance components and the prediction of genetic trends (Legarra *et al.* 2015). Founder relationships can be estimated from marker data of genotyped individuals (Christensen 2012; Legarra *et al.* 2015; Colleau *et al.* 2017), which are usually only available for younger generations in ongoing breeding programs. In the context of mapping of quantitative trait loci (QTL) in line-cross experiments inferences on within-line relationships between QTL-genotypes of founders can also be made from variance components and related likelihoods (Rönnegård *et al.* 2008).

In the context of crossbreeding, founders comprise individuals from two or more genetically distinct populations. This requires relationship coefficients for each single population, in addition to a combination of populations (Legarra *et al.* 2015). Here, the aim is to model relationships between purebreds and also between purebreds and crossbreds, most frequently from the F_1_ generation. Applications are in genetic evaluations, were purebred and crossbred performances are treated as genetically correlated traits (Aguilar *et al.* 2010; Pszczola *et al.* 2012; de los Campos *et al.* 2013; Garcia-Baccino *et al.* 2017). For this purpose, the interest is in the genetic (co-)variance components for these traits in the purebred populations, where selection takes place.

A somewhat different focus exists when composite populations are generated, *e.g.*, for selection experiments in laboratory animals (Holt *et al.* 2005) or when building advanced intercross lines (AIL, Darvasi and Soller 1995) for fine-mapping purposes. In this case, two or more genetically distinct lines, in some cases inbred lines, are intercrossed. The population is further developed from generation to generation by inter-mating crossbreds. Only performance traits of the intercross are entered into the analyses of, for example, selection experiments. This is an undertaking for which the use of mixed models, with an appropriate relationship matrix, has been recommended (Walsh and Lynch 2018, p. 631-668). The genetic variance in the intercross generations later than F_1_ includes the so-called segregation variance (Lande 1981; Lo *et al.* 1993), which is caused by loci that are fixed for different alleles in the founder lines but begin to segregate from the F_2_ generation onwards. The proportion to which the segregation variance contributes to the total genetic variance in the F_2_ and later generations can, in principle, vary between zero and one. However, this proportion cannot be derived from pedigree data alone.

As a solution, we propose a relationship matrix that takes account of known marker allele frequencies of founders. Those markers that are fixed for alternative alleles in different lines largely determine the extent of the role of segregation variance at an average locus. Rules for the Mendelian transmission of these relationships to later generations were derived for both autosomal and X-chromosomal relationships. These matrices can then be combined with information on observed genotypes, which may include non-founders, and be used for the estimation of variance components and genetic trends. The associated genetic variance is thereby defined as the variance among unrelated individuals in the first generation of the composite population (*i.e.*, the F_2_ in a two line cross). In addition, an application is presented to obtain estimates of genetic paramters for litter size and growth in an advanced intercross between a long-term selected, high fecundity mouse line and a highly inbred control line.

## Theory

### Underlying assumptions

We assume two distinct founder populations; A and B, that contribute to a composite crossbred population. All founders are assumed to be genotyped and line specific founder allele frequencies are known. For each marker i, founder frequencies are denoted as $p_{i}^{A}$ and $p_{i}^{B}$. Under the condition that all founders contribute equally to the composite population, the expected allele frequency in the F_2_ generation ${\bar{p}}_{i}$, is fully determined as the average, p¯i=12(piA+piB), of the two line specific frequencies.

### Observed genomic founder relationships

The genotypes of the founders can be summarized into a centered genotype matrix, $Z_{0}$, with one row per individual and one column per marker.

#### Autosomes:

For autosomal markers, entries into the $Z_{0}$ matrix are $z_{i}^{k}\in (2-2{\bar{p}}_{i}\text{, }1-2{\bar{p}}_{i}\text{, }0-2{\bar{p}}_{i})$ for genotypes AA, Aa and aa, respectively. The observed genomic relationship matrix, $G_{0}$, between founders is

$$G_{0}=Z_{0}Z_{0}^{\prime }/S$$ (1)

where S is the scaling factor; $S=2\sum_{i}{\bar{p}}_{i}(1-{\bar{p}}_{i})$. $G_{0}$ is a standard genomic relationship matrix, except when using ${\bar{p}}_{i}$ for centering and scaling, as previously described by Van Raden (2008).

#### X-chromosome:

The observed X-chromosomal genomic relationship matrix is set up in accordance with the rules for autosomal markers. Extra details apply to the definitions of average gene frequencies and the treatment of male (hemizygous) individuals. For X-chromosome markers we define the mean allele frequency ${\bar{p}}_{i}$, again, as p¯i=12(piA+piB). However, on the X-chromosome this is only equal to the gene frequency to be ultimately reached in later generations if the two founder lines contribute equally through males and females to the genetic makeup of the composite population.

Genotype codes have to be transformed to gene counts; $c_{i}^{k}\in (1,0)$, for male founders and then centered by ${\bar{p}}_{i}$ instead of $2{\bar{p}}_{i}$. For matrix $Z_{0}$ the entries for X-chromosome markers are $z_{i}^{k}\in (1-{\bar{p}}_{i}\text{, }0-{\bar{p}}_{i})$, for genotypes A and a, respectively. The X-chromosome genotypes for female founders are, in contrast, treated in the same way as autosomal markers. The observed X-chromosomal genomic relationship matrix can then be calculated by equation (1), using X-chromosomal gene counts and the scaling factor S, as defined above.

### Expected founder genomic relationships

#### Autosomes:

An expectation of $G_{0}$ (denoted $\text{E}(G_{0})$) can be derived under the assumption that alleles at independent loci are randomly sampled from each founder line’s particular gene pool (Binomial sampling), as defined by their known founder allele frequencies. We consider two gametes randomly chosen from base population A. The 2×2 matrix $R^{AA}$ has the expected sum of squared centered coefficients $z_{i}^{A}$ ($z_{i}^{A}\in (1-{\bar{p}}_{i},0-{\bar{p}}_{i})$, for alleles A and a, respectively) for each of the gametes on its diagonal and the corresponding expected sum of cross products on its off diagonal,

$$R^{AA}=\left[\begin{array}{cc} d^{AA} & r^{AA}\\ r^{AA} & d^{AA} \end{array}\right]=\frac{1}{S}[\begin{array}{cc} \sum_{i}\left[p_{i}^{A}(1-p_{i}^{A})+{\left(p_{i}^{A}-{\bar{p}}_{i}\right)}^{2}\right] & \sum_{i}{\left(p_{i}^{A}-{\bar{p}}_{i}\right)}^{2}\\ \sum_{i}{\left(p_{i}^{A}-{\bar{p}}_{i}\right)}^{2} & \sum_{i}\left[p_{i}^{A}(1-p_{i}^{A})+{\left(p_{i}^{A}-{\bar{p}}_{i}\right)}^{2}\right] \end{array}].$$ (2)

For base population B, the equivalent matrix $R^{BB}$ can be derived from its line specific allele frequencies $p_{i}^{B}$. In the following matrices, $R^{AA}$ and $R^{BB}$ are referred to as the expected covariance matrices between gametes from the same founder line.

Furthermore, we can set up $R^{AB}$ as the equivalent relationship matrix of two randomly chosen gametes from populations A and B:

$$R^{AB}=\left[\begin{array}{cc} d^{AA} & r^{AB}\\ r^{AB} & d^{BB} \end{array}\right]=\frac{1}{S}[\begin{array}{cc} \sum_{i}\left[p_{i}^{A}(1-p_{i}^{A})+{\left(p_{i}^{A}-{\bar{p}}_{i}\right)}^{2}\right] & \sum_{i}\left(p_{i}^{A}-{\bar{p}}_{i})(p_{i}^{B}-{\bar{p}}_{i}\right)\\ \sum_{i}\left(p_{i}^{A}-{\bar{p}}_{i})(p_{i}^{B}-{\bar{p}}_{i}\right) & \sum_{i}\left[p_{i}^{B}(1-p_{i}^{B})+{\left(p_{i}^{B}-{\bar{p}}_{i}\right)}^{2}\right] \end{array}].$$ (3)

From the distinct elements in $R^{AA}$ and $R^{AB}$ (also $R^{BB}$) we can compute all necessary expected relationships between individuals, which may occur in $\text{E}(G_{0})$. First, we have the expected self-relationship of an individual from a particular founder line (*e.g.*, line A)

$$\omega^{A}=2d^{AA}+2r^{AA}.$$ (4)

The expected relationship between two individuals from the same founder line is

$$\omega^{AA}=4r^{AA}.$$ (5)

The expected relationship between two individuals from two different founder lines is

$$\omega^{AB}=4r^{AB}.$$ (6)

#### X-chromosome:

With regard to expected X-chromosomal relationships, different combinations between either males or females may occur, in addition to the same or different founder lines. In total, there are eight different kinds of possible relationships: the self-relationship of a male originating from a certain line, $\omega_{x}^{AA}$; the expected relationship between two males from the same line, $\omega_{x,x}^{AA}$, or $\omega_{x,x}^{AB}$ from different lines; the self-relationship, $\omega_{xx}^{AA}$, of a female from a certain line; the relationships $\omega_{xx,xx}^{AA}$ and $\omega_{xx,xx}^{AB}$ between two females from the same line and different lines, respectively, and finally the relationships $\omega_{x,xx}^{AB}$ and $\omega_{xx,x}^{AB}$ between a male and a female from different lines. Formulas for all eight cases are summarized in Table 1.

**Table 1:** Formulas for calculating the different types of expected X-chromosomal relationships of males and females in a crossbred population derived from two founder lines A and B

	Relationship*^a^*	Formula*^b^*
Self-relationship	$\omega_{x}^{A}$	1S∑i[piA(1−piA)+(piA−p¯i)2]=dA
	$\omega_{xx}^{A}$	$2\omega_{x}^{A}+2\omega_{x,x}^{AA}=2d^{A}+2r^{A}$
Relationships between individuals	$\omega_{x,x}^{AA}$	1S∑i[(piA−p¯i)2]=rA
	$\omega_{x,x}^{AB}$	1S∑i[(piA−p¯i)(piB−p¯i)]=rAB
	$\omega_{xx,xx}^{AA}$	$4\omega_{x,x}^{AA}=4r^{A}$
	$\omega_{xx,xx}^{AB}$	$r^{A}+r^{B}+2r^{AB}$
	$\omega_{x,xx}^{AA}$	$2r^{A}$
	$\omega_{x,xx}^{AB}$or $\omega_{xx,x}^{AB}$	$2r^{AB}$

a $\omega_{x}^{A}$ and $\omega_{xx}^{A}$ are the expected self-relationships of male and female, respectively; $\omega_{x,x}^{AA}$ and $\omega_{x,x}^{AB}$are the expected relationships between two males, which are all from line A, or from different lines (A and B respectively); $\omega_{xx,xx}^{AA}$ and $\omega_{xx,xx}^{AB}$are the expected relationships between two females, which are all from line A, or from different lines (A and B respectively); $\omega_{x,xx}^{AA}$, $\omega_{x,xx}^{AB}$ and $\omega_{xx,x}^{AB}$ are the expected relationships between a male and a female, which are all from line A, or from different lines (A and B respectively).

b $p_{i}^{A}$ and $p_{i}^{B}$are the line-specific allele frequencies of lines A and B for locus i; ${\bar{p}}_{i}$ is the mean of $p_{i}^{A}$ and $p_{i}^{B}$ for locus i; $d^{A}$ and $d^{B}$ are the expected self-relationships of gametes inherited from line A, or from line B; $r^{A}$, $r^{B}$ and $r^{AB}$ are the expected relationships between two gametes, that are from the same line (line A or line B) or from different lines.

### Extending expected genomic relationships to later generations

Expected founder genomic relationships can be extended to all descendants by following the paths of Mendelian transmission, as specified in the pedigree. The resulting expected genomic relationship matrix is denoted by Ã. The diagonal elements $g_{k,k}^{E}$ and off-diagonal elements $g_{k1,k2}^{E}$ of matrix Ã are computed by a modified version of the tabular method (Emik and Terrill 1949; Cruden 1949).

#### Autosomes:

The expected autosomal self-relationships (diagonal elements of Ã) consist of three parts; the expected self-relationships of gametes inherited from the sire, from the dam and the gametic relationship between these parental gametes. Relationships between individuals (off-diagonal elements) are an average of the relationships of one candidate and the parents of another, as known from the tabular method. The expected self-relationships and relationships are:

$$g_{k,k}^{E}=s_{sire}+s_{dam}+\frac{1}{2}g_{sire,dam}^{E}$$ (7)

$$g_{k1,k2}^{E}=\frac{1}{2}(g_{sire1,k2}^{E}+g_{dam1,k2}^{E})$$ (8)

where sire and dam are the parents of individual k, sire1 and dam1 are the parents of individual k1, $s_{sire}$ and $s_{dam}$ are the expected self-relationships of gametes that individual k inherits from their parents (see supplement for a derivation of equations 7 and 8).

These formulas are applicable from the F_1_ generation onwards, whereby their components depend on generation number. For F_1_ individuals $s_{sire}=\frac{1}{2}(2d^{AA})=d^{AA}$and $s_{dam}=\frac{1}{2}(2d^{BB})=d^{BB}$ if we assume a male founder from line A and a female founder from line B. The $g_{sire,dam}^{E}$ is the expected relationship between two founders, *i.e.*, the parents of an F_1_ individual, which in our case gives $g_{sire,dam}^{E}=\omega^{AB}=4r^{AB}$. Generally, for an F_1_ individual the expected self-relationship is $g_{k,k}^{E}=d^{AA}+d^{BB}+2r^{AB}$. Individuals from the F_2_ generation receive gametes from each parent with a 50% probability for line A and line B alleles. Therefore, in the F_2_, $s_{sire}=\frac{1}{2}(d^{AA}+d^{BB})$ and $s_{dam}=\frac{1}{2}(d^{AA}+d^{BB})$.

With the two founder lines used in our case, the sum of $d^{AA}$ and $d^{BB}$ is equal to 1 but $d^{AA}$ and $d^{BB}$ are usually different (see supplement). The expected self-relationship for a gamete that an F_2_ individual inherits from one of its parents is always equal to 0.5 and $g_{k,k}^{E}=1+\frac{1}{2}g_{sire,dam}^{E}$. The same applies to later generations.

#### X-chromosome:

For the X-chromosome, equations (7) and (8) are modified for females to give

$$g_{k,k}^{E}=s_{sire}+s_{dam}+g_{sire,dam}^{E}$$ (9)

$$g_{k1,k2}^{E}=g_{sire1,k2}^{E}+\frac{1}{2}g_{dam1,k2}^{E}$$ (10)

and for males to give

$$g_{k,k}^{E}=s_{dam}$$ (11)

$$g_{k1,k2}^{E}=\frac{1}{2}g_{dam1,k2}^{E}.$$ (12)

Note that Fernando and Grossman (1990) introduced similar equations for calculating the pedigree-derived relationships for the X-chromosome, were the self-relationship for males, $s_{sire}$, is 0.5 in all generations, meaning $s_{dam}$ must also be 0.5. The underlying assumption is that allele frequencies of both sexes are equal, as it is the case if the population is in an equilibrium state (Self and Liang 1987). Equations (9) to (11), in contrast, allow $s_{sire}$ and $s_{dam}$ to fluctuate along with male and female marker frequencies in early generations, after mating male and female founders with differing allele frequencies.

### Expected genomic co-variances in an infinitely large F_2_ population

In a cross between two populations, A and B, the gametes that an individual receives in the F_2_ generation are of types A and B, with equal probability. Such an individual will have a probability of 0.25 of inheriting two A gametes, a probability of 0.25 of inheriting two B gametes and a probability of 0.5 of inheriting one A gamete and one B gamete.

The average self-relationship of an individual in an infinitely large F_2_ population is, therefore

$$\begin{array}{c} \frac{1}{4}\left(2d^{AA}+2r^{AA}\right)+\frac{1}{4}\left(2d^{BB}+2r^{BB}\right)+\frac{1}{2}\left(d^{AA}+d^{BB}+2r^{AB}\right)\\ =\frac{1}{4}\left(2d^{AA}-2r^{AB}\right)+\frac{1}{4}\left(2d^{BB}-2r^{AB}\right)+\frac{1}{2}\left(d^{AA}+d^{BB}+2r^{AB}\right)\\ =d^{AA}+d^{BB}\\ =1 \end{array}$$

as $r^{AA}=r^{BB}=-r^{AB}$ in a cross of two lines.

The average covariance between F_2_ individuals can be derived as the weighted average of covariances in nine possible combinations of two individuals, were each of them may carry two (AA), one (AB) or no (BB) gametes from the A line. These nine single pair-wise covariances can be expressed in terms of relationships between gametes, *i.e.* $r^{AA}$, $r^{BB}$ and $r^{AB}$. The weights are the probabilities of the occurrence of all these combinations:

$$\begin{array}{l} \frac{1}{16}4r^{AA}+\frac{2}{16}\left(2r^{AA}+2r^{AB}\right)+\frac{1}{16}2r^{AB}\\ +\frac{2}{16}\left(2r^{AA}+2r^{AB}\right)+\frac{4}{16}\left(r^{AA}+r^{BB}+2r^{AB}\right)+\frac{2}{16}\left(2r^{BB}+2r^{AB}\right)\\ +\frac{1}{16}4r^{AB}+\frac{2}{16}\left(2r^{AB}+2r^{BB}\right)+\frac{1}{16}4r^{BB}=0. \end{array}$$

The expected covariance matrix Ã for unrelated and non-inbred F_2_ individuals is, therefore, an identity matrix. This means that such a hypothetical population can be viewed as a reference for the actual composite population derived from the genotyped founders. Variance components estimated with an Ã matrix can, after proper adjustment for founder relationships (Legarra *et al.* 2015), be interpreted as the genetic variance in such a population. When defined in this way the genetic variance includes the segregation variance, *i.e.*, the difference between the genetic variance in the F_2_ and the F_1_ (Lande 1981; Lo *et al.* 1993). The segregation variance can be expressed as function of the self-relationships of non-inbred individuals in the F_2_ and the F_1_ generations:

$$\left[1-\left(d^{AA}+d^{BB}+2r^{AB}\right)\right]\sigma_{a}^{2}=-2r^{AB}\sigma_{a}^{2}.$$

Note that in the case of a cross between two inbred lines $d^{AA}=d^{BB}=-r^{AB}=\frac{1}{2}$ and the last formula correctly flags all genetic variance as segregation variance.

### Accounting for observed genotypes

#### Combined relationship matrix H:

Expected genomic founder relationships will generally differ from those observed. This can be taken into account by applying a previously developed theory (Legarra *et al.* 2009; Christensen and Lund 2010; Aguilar *et al.* 2010) for combining pedigree-derived relationships (A) and genomic relationships into a joint matrix, H. In our case we used H as a modification of Ã that is corrected for the observed founder relationships in $G_{0}$. We denote Ã as the expected genomic relationships of founders. In terms of the inverse of H (Christensen and Lund 2010; Aguilar *et al.* 2010), we then get

$$H^{-1}={Ã}^{-1}+\left[\begin{array}{cc} G_{0}{}^{-1}-{Ã}_{11}^{-1} & 0\\ 0 & 0 \end{array}\right]={Ã}^{-1}+\left[\begin{array}{cc} G_{0}{}^{-1}-\text{E}{\left(G_{0}\right)}^{-1} & 0\\ 0 & 0 \end{array}\right].$$

The inverse of matrix Ã is described in the supplement. The observed founder genomic relationship matrix, $G_{0}$, may be singular. In this case, one may capitalize on the idea of blending (Garcia-Baccino *et al.* 2017). We used $G_{0}^{BLD}=0.98\times G_{0}+0.02\times \text{E}\left(G_{0}\right)$, instead of $G_{0}$, when computing $H^{-1}$.

#### Joint relationships from simulated genotypes:

For the sake of comparison with H, a joint relationship matrix, G, was generated from observed genotypes of founders plus simulated genotypes of non-founders. The alleles were randomly sampled from observed founder genotypes and simulated marker genotypes of offspring in later generations were derived by gene-drop. The expected self-relationships for autosomes were calculated as

$$g_{k1,k2}^{E}=\frac{1}{S}\sum_{i}\text{E}[(c_{i}^{k1}-2{\bar{p}}_{i})(c_{i}^{k2}-2{\bar{p}}_{i})]$$ (13)

where $g_{k1,k2}^{E}$ is the expected relationship between individuals k1 and k2, $c_{i}^{k1}$ and $c_{i}^{k2}$ are the gene counts of individuals k1 and k2 at locus i. For self-relationship we used gk,kE=1S∑iE[(cik−2p¯i)2]. Expectations were obtained by averaging over 10000 replicates of the gene-drop simulation.

For X-chromosomes, the sex of the descendants must also be considered. For female descendants equation (13) remains. For male descendants equation (13) will be modified by applying the centered X-chromosomal genotypes ($z_{i}^{k}\in (1-{\bar{p}}_{i},0-{\bar{p}}_{i})$), which gives gk.kE=1S∑iE[(cik−p¯i)2].

## Application Example

### Animals, pedigree, phenotypes and genotypes

The advanced intercross mouse line (AIL) bred in the Leibniz Institute for Farm Animal Biology (FBN) was established by randomly choosing and intercrossing four females from the long-term selected, high-fecundity line, FL1 (Langhammer *et al.* 2014), and four males from a highly inbred (theoretical inbreeding coefficient > 0.999) control line, DUKsi (Alm *et al.* 2010). Both lines were derived from the same initial gene pool (Dietl *et al.* 2004).

The high fecundity FL1 line was selected for an index trait that combines litter size (LS0) and litter weight (LW0) at birth in primiparous females (Index I = 1.6 × LS0 + LW0) up to generation 131. As a result of selection over 131 generations an average of 17.14 ± 3.25 pups per litter had been reached. This is a 1.8 fold higher fecundity than observed in the control line (see Table 2). An outbred control line, DUKs, was maintained at approximately the same population size for 79 generations by random mating and without any selection pressure. The inbred derivative DUKsi was split from DUKs in generation 79.

**Table 2:** Mean & SD of litter size and litter weight of two founder lines

Line	Generation	N	LS0 (mean ± SD)	LW0 (mean ± SD)
FL1	121-131	730	17.14 ± 3.25	27.27 ± 5.03
DUKsi	28-38	753	9.84 ± 2.08	14.74 ± 2.82

To evaluate the difference of LS0 and LW0 between controls and selected mice reference animals were collected from 10 generations (around 70 litters per generation) from FL1 and control line DUKsi, respectively.

Four male founders were chosen for the experiment after 38 generations of full sib mating in the DUKsi line. Each of four females from generation 131 of the FL1 selection line was mated with one male from the control line. The F_1_ litters were standardized to four male and eight female pups immediately after birth in order to maintain a surplus of females for further reproduction. Full sibs from the four initial F_1_ families were then repeatedly (at least four times) inter-mated by rotating males and females within the family. Thus, each of the four pairs of founder parents constitutes a family of its own, with descendants up to generation F_3_. Offspring of only one of these families were then maintained and became the ancestors of all further generations of the AIL.

A total of 19266 mice (9453 males and 9813 females) were used for this study. They were distributed unevenly across all generations; 44 in F_1_, 1483 in F_2_, 5235 in F_3_, 1025, 1058 and 1070 in F_23_, F_24_ and F_25_, respectively, and between 312 and 431 for other generations.

Reproductive ability was measured as litter size at birth (LS0) and litter weight at birth (LW0). The litter traits were recorded for 4430 females (from 9813 females) for their first litter. Among these females, 1481 also had a record for their second litter (there were no second litter records for generations from F_3_ to F_21_). Growth traits that were recorded for all generations were body weight at day 21, 42 and 63 (BM21, BM42, BM63), in addition to body weight at first mating (BMM).

The six fertility and growth traits (see Table 3 for summary statistics) were analyzed using different kinds of relationship matrices, as described below.

**Table 3:** Descriptive statistics for traits related to litter size and growth. Litter size and litter weight was recorded in first (LS0_1, LW0_1) and partially also in second (LS0_2, LW0_2) parity females. Body mass of both sexes was measured at days 21 (BM21), 42 (BM42), 63 (BM63) after birth as well as at day of mating (BMM)

Trait	N	Generation*^a^*	Mean (SD)
LS0_1	4,430	F_2_-F_29_	16.025 (3.286)
LS0_2	1,481	F_2_, F_22_-F_25_	16.315 (3.766)
LW0_1	4,410	F_2_-F_29_	27.456 (4.807)
LW0_2	1,474	F_2_, F_22_-F_25_	28.627 (5.530)
BM21	19,080	F_2_-F_30_	10.878 (2.013)
BM42	14,586	F_2_-F_30_	29.439 (3.787)
BM63	13,910	F_2_-F_30_	35.185 (3.801)
BMM	7,416	F_2_-F_29_	36.418 (4.167)

a Generation, generations from which phenotypic records were available. For instance, for trait LW0_1 data were recorded from generations from F_2_ to F_29_ and for trait LW0_2 data were only recorded from five generations F_2_, F_22_, F_23_, F_24_ and F_25_.

All eight founders of this intercross line were genotyped with the JAX Mouse Diversity Genotyping Array (Yang *et al.* 2009) at the genotyping facility of the Jackson Laboratory, The Jackson Laboratory, Bar Harbor, Maine, USA.

### Comparative estimation of variance components

Two fertility and four growth traits were comparatively analyzed with mixed models that comprised of different kinds of relationship matrices. Fertility traits (LS0, LW0) were analyzed as traits of females (Langhammer *et al.* 2017). For both traits, the model for the i_th_ observation (i=1,2) of animal a was

$$y_{gacpi}=\mu +\beta_{g}+\gamma w_{a}+a_{a}+x_{a}+u_{c}+u_{p}+e_{gacpi}$$ (14)

where $\beta_{g}$ is the fixed generation effect for females born in generations from F_2_ to F_29_ (g = 1, ... 28) and γ is the linear regression of the body weight *w_a_* at mating of each female’s mother. The random part of the model comprised the additive autosomal and X-chromosomal genetic effects $a_{a}$ and $x_{a}$ of animal a (a=1, ..., 19266), the common litter environmental effect $u_{c}$ (c=1, ..., 2420), the permanent environmental effect $u_{p}$ (p=1, ..., 4430) and the residual $e_{gacpi}$. The covariance matrices of the common litter environmental and permanent environmental effects were assumed as equal to an identity matrix of proper size times the respective variance component.

Observations of growth traits (BM21, BM42, BM63, and BMM) were made from males and females from generations F_2_ to F_30_. Therefore, the fixed part of the model also included an additional sex effect, $\beta_{s}$ (s=1,2), and the number of levels was 29 for the generation effect. For BM21 and BMM no permanent environmental effect could be fitted because these traits were only measured once for all animals. Table 4 gives more details of the model we applied.

**Table 4:** Numbers of observations (N) and number of levels for fixed and random effects in models fitted to litter size (LS0), litter weight (LW0) and body mass traits at ages of 21, 42, 63 days and at mating (BM21, BM42, BM63, BMM). Fixed effects are generation number, linear regression on mother’s weight at mating, litter number and sex; random effects are additive genetic, litter and permanent environmental effects

Trait	N	Generation	Weight of Mother	Litter Number	Sex	Additive Genetic Effect	Litter env.	Permanent env.
LS0	5,911	28	1	2	1	4,430	2,420	1481
LW0	5,884	28	1	2	1	4,410	2,397	1474
BM21	19,080	29	1	—	2	19,080	4,362	—
BM42	14,586	29	1	—	2	13,959	3,132	627
BM63	13,910	29	1	—	2	13,283	3,044	627
BMM	7,416	28	1	—	2	7,416	2,626	—

All random effects were assumed to be mutually independent. Three different kinds of relationship matrices were compared for autosomal and X-chromosomal genetic effects: pedigree-derived relationship matrices that assumed unrelated founders, H matrices, as explained above, and G matrices, based on gene-drop simulations (denoted as “A”, “H” and “G”, respectively). Model variants “A_a_”, “G_a_” and “H_a_” include only an autosomal relationship matrix of one of the specified types. Model variants “A_a+x_”, “G_a+x_” and “H_a+x_” additionally include the X-chromosomal relationship matrix of the same type. The Restricted maximum likelihood (REML) estimates of all variance components were obtained from the ASReml package (Gilmour *et al.* 2015). All estimated genetic variance components from model variants, which included founder relationships, were corrected for non-independence of founders by multiplication with a correction factor, $D_{k}$ (Searle 1982. p. 355; Legarra 2016):

$$D_{k}=\frac{\sum_{i=1}^{f}g_{0}\left(i,i\right)}{f}-\frac{\sum_{i=1}^{f}\sum_{j=1}^{f}g_{0}\left(i,j\right)}{f^{2}}$$

where $g_{0}\left(i,j\right)$ are elements of either the observed founder relationship matrix, $G_{0}$ (after blending), or a respective diagonal matrix for pedigree-derived relationships, and f=8 is the number of founders.

The significance of X-chromosomal genetic effects was evaluated by comparing the full model for each trait with a reduced model without X-chromosomal genetic effects. Error probabilities were derived via restricted likelihood ratio tests (RLRT), with a single degree of freedom (Wiencierz *et al.* 2011).

### Animal welfare declaration

The animal experiments were performed following national and international guidelines and were approved by the local authorities (Landesamt für Landwirtschaft, Lebensmittelsicherheit und Fischerei, Mecklenburg-Vorpommern, Germany).

### Data availability

Marker genotypes of founders, a pedigree file for all AIL animals and a data file with observed phenotypes for the six analyzed traits can be found in the RADAR (research data repository) repository under https://doi.org/10.22000/88. An R-program that sets up the Ã matrix for a two-line cross according to the rules explained above (autosomal and X-chromosomal) is available from the first author. Supplemental material available at Figshare: https://doi.org/10.25387/g3.7110440.

## Results and Discussion

### Founder relationship matrices

#### Marker data:

The number of polymorphic autosomal SNPs (single nucleotide polymorphisms) was 140,532 in all founders (see Table 5). The number that segregated in the FL1 line alone was 44,827, while 67,450 segregated within the DUKsi control line. The numbers on the X-chromosome (non-pseudoautosomal, Perry *et al.* 2001) were 2,009 for all founders, 191 in the FL1 line and 1,055 in the control line. Opposite alleles with frequencies at hundred and zero per cent in the two lines (line-specific alleles) occurred with 38.9% on autosomes as well as the X-chromosome. Polymorphic markers were evenly distributed across the genome (see Figure S1 in supplement file) and the density (number per 1 Mbp) was between 34.0 and 70.4 (see Table S1 in supplement file).

**Table 5:** Numbers of SNP-markers which were segregating within all eight founders, and within both founder lines (FL1, control). Line-specific SNPs do not segregate within founders of the same line but have alternative kinds of alleles in each line

ChromosomalLocation	All Founders	FL1	Control	Line-specific SNP
autosomal	140,532	44,827	67,450	54,654
X-chromosomal	2,009	191	1,055	869

#### Observed genomic relationships:

**Figure 1:**
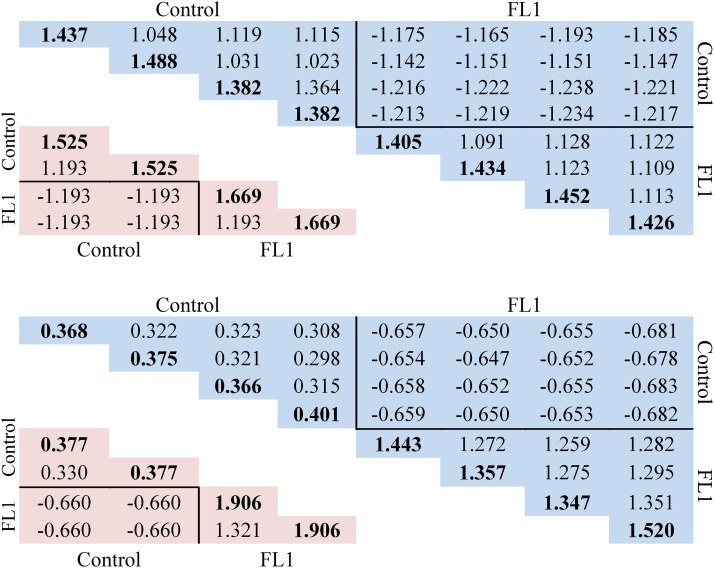
Comparison of the observed and expected founder genomic relationship matrices. The upper panel shows the autosomal observed genomic founder relationship matrix **G** (marked in blue) and the autosomal expected founder relationship matrix **G**^**E**^ (marked in red). The lower panel shows X-chromosomal **G** (marked in blue) and X-chromosomal **G**^**E**^ (marked in red). Diagonal elements are in bold.

The observed 8×8 genomic founder relationship matrices are shown in Figure 1 as triangular matrices for autosomes (above) and X-chromosomes (below). Observed autosomal self-relationships were fairly uniform in both the control (between 1.382 and 1.488) and the FL1 line (between 1.405 and 1.452). The expected self-relationships (lower triangle of 4×4 matrix, same panel) were 1.525 and 1.669 for the control and FL1 founders, respectively. The lower observed relationships (approximately 7% in controls and 14% in the FL1 line) indicate an excess of heterozygosity in both lines relative to within line Hardy-Weinberg proportions at observed allele frequencies. These deviations can be explained by the sampling of rare alleles, which are more likely to occur in a heterozygous condition when compared with more frequent alleles. Observed self-relationships are, however, considerably larger than one due to elevated homozygosity relative to the non-inbred F_2_ individuals that define the base population. Despite some fluctuations, relationships between founders of the same line (expected: 1.193) and different lines (expected: -1.193) barely deviate from the expectations. On the X-chromosome, the observed self-relationships of female control founders (range: 1.347 - 1.520) deviate more (about 26%) from the expectation of 1.906, while in contrast, observed self-relationships of male control founders and all observed X-chromosomal relationships between male and female founders agree well with the expectations. On both kinds of chromosomes, large negative relationships between individuals from different lines are predominantly a result of SNPs with line-specific alleles. Their high proportion and the resulting negative between-line relationships reflect the long lasting separation of the two lines and their selection for different goals. Consequently, the expectation of $\omega^{AB}=4r^{AB}=1.193$ translates into a proportion of $-2r^{AB}=2\cdot 0.29825\approx 0.6$ of the total genetic variance that can be attributed to segregation variance.

Both the autosomal and X-chromosomal observed genomic relationship matrices are singular, with rank seven. In the case of the autosomal markers, this is caused by the relationship of 1.36 between the third and fourth founders ($g_{3,4}$), which is close to the self-relationship of the same animals (1.38). This translates into a very close correlation of almost one. The background is that the inbred control line actually consisted of several sublines that can be traced back to the same pair of ancestors. Sublines were generated by branching the main line in different generations and maintained by repeated full-sib mating. Unintentionally, two male founders were sampled from the same subline and the other male founders from two different sublines. In the case were all control founders had been drawn from the same subline, the rank for the observed relationship matrix is expected to be five, as almost no genetic variation is expected within the subline.

As a consequence of this rank deficiency, the observed relationship matrices are not invertible. This was solved by blending them with their expected counterparts (see Theory). Alternatively, one could have averaged the two columns and rows of the highly correlated animals and used this average as a replacement in a 7×7 relationship matrix, thereby assigning a single genetic effect to both founders (*e.g.*, Tuchscherer *et al.* 2004).

#### Evolution of self-relationships over generations:

**Figure 2:**
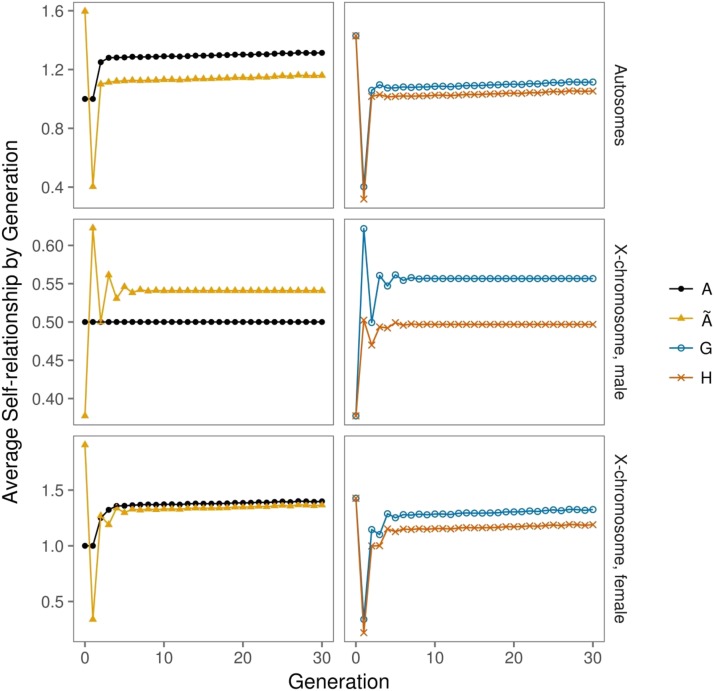
Comparison of the generation mean of the self-relationships for the relationship matrices derived in the study. A is the pedigree-derived relationship matrix. Ã is the relationship matrix derived from the pedigree and the allele frequencies of all founder lines. G is the pedigree-genotype-combined relationship matrix derived by “gene drop”. H is the relationship matrix derived by Legarra’s method (2008) using matrix Ã instead of matrix A. The X-chromosomal self-relationships are divided by sex. The oscillatory approach of the allele frequency of the X-linked markers can be observed in the first few generations of the curves for matrices Ã, G and H, when compared with the matrix A and the autosomal cases.

The mean self-relationships, as derived from different kinds of relationship matrices, develop differently over generations (Figure 2). The classical pedigree-derived matrix A has diagonal elements of one in the base generations and the F_1_, followed by a jump to 1.25, which indicates inbreeding of F_2_ animals due to full-sib mating in the F_1_. From then on there is only a very slight increase of the mean inbreeding coefficient. This pattern is present for autosomal relationships (Figure 2, upper left), as well as for X-chromosomal self-relationships in females (lower left panel). Self-relationships from Ã, in contrast, show strong fluctuations from high position values in founders to high negative values in the F_1_ (same panels). Autosomal self-relationships from the Ã matrix reach an average of larger than one in the F_2_, which increases only slightly in further generations (upper left panel). The Ã matrix is scaled in such a way that non-inbred F_2_ animals would have a self-relationship of one. Therefore, larger values are a sign of a higher expected homozygosity when compared with this reference population. The fluctuations of average generational autosomal self-relationships come to an end from generation F_2_ onwards (upper left), while they continue, albeit with decreasing amplitudes, for X-chromosomal self-relationships in males (middle left) and females (lower left). The underlying reason is that the genetic equilibrium is reached after two generations for autosomal markers, when initial allele frequencies differ in males and females (Crow and Kimura 1970). This process takes longer for X-chromosomal loci (Li 1976, p. 137). In line with this, the amplitudes for male X-chromosomal self-relationships have the opposite sign to those for females.

The mean X-chromosomal self-relationships of males stabilize at around 0.54, which is somewhat larger than 0.5. The reason is that the actual equilibrium allele frequencies approach ${\bar{p}}_{i}=\frac{2}{3}p_{i}^{FL1}+\frac{1}{3}p_{i}^{control}$ instead of ${\bar{p}}_{i}=\frac{1}{2}(p_{i}^{FL1}+p_{i}^{control})$, since all founders from the FL1 line were females with two alleles and all founders from the control line had only a single allele at each X-chromosome locus. The X-chromosomal Ã matrix was, however, computed under the assumption of equal contributions of both founder lines to the F_2_, which would require equal numbers of male and female founders from both lines. Values of 0.54 therefore indicate somewhat more X-chromosomal variability, as in a reference population were ${\bar{p}}_{i}=\frac{1}{2}(p_{i}^{FL1}+p_{i}^{control})$.

Average self-relationships from H and G types of relationship matrices can be seen on the panels to the right of Figure 2. The initial fluctuation patterns already described for Ã are also present in the average self-relationships of these two matrices. The three curves for the G matrices are similar but not identical to Ã. In comparison, averages for the H matrices are lower in all cases, which is a result of correcting Ã to lower observed homozygosity than expected, under the assumptions made for the construction of Ã.

#### Genetic parameters:

The genetic variance components and heritability for six selected traits can be found in the Table 6 and Table S2. The X-chromosomal genetic variance proved to be significant at the 5% level for the three growth traits; BM21, BM42 and BM63, regardless of what type of relationship matrix was used as part of the model (Table S3). The additive genetic variance component for the sex chromosome was almost zero for BMM (see Table S2) and was also not significant for litter traits LS0 and LW0 (Table S3).

**Table 6:** Estimates of genetic parameters for all traits obtained by three different kinds of relationship matrices

Trait	Relationship	$\sigma_{a}^{2}$	$\sigma_{x}^{2}$	$\sigma_{c}^{2}$	$\sigma_{p}^{2}$	$\sigma_{e}^{2}$	$h^{2}$
LS0	A_a_	1.37 (0.30)		0.31 (0.16)	2.06 (0.33)	7.79 (0.27)	0.12 (0.03)
	G_a_	2.25 (0.42)		0.30 (0.16)	1.76 (0.34)	7.81 (0.27)	0.19 (0.03)
	H_a_	2.44 (0.45)		0.31 (0.16)	1.70 (0.34)	7.81 (0.27)	0.20 (0.03)
LW0	A_a_	5.76 (0.83)		0.53 (0.33)	3.86 (0.70)	14.5 (0.5)	0.23 (0.03)
	G_a_	8.46 (1.10)		0.49 (0.33)	3.15 (0.72)	14.5 (0.5)	0.32 (0.03)
	H_a_	9.09 (1.17)		0.50 (0.33)	3.02 (0.73)	14.6 (0.5)	0.34 (0.04)
BM21	A_a+x_	0.54 (0.07)	0.02 (0.01)	2.15 (0.06)		0.61 (0.03)	0.17 (0.02)
	G_a+x_	0.73 (0.09)	0.02 (0.01)	2.13 (0.06)		0.59 (0.03)	0.22 (0.02)
	H_a+x_	0.77 (0.10)	0.03 (0.02)	2.12 (0.06)		0.59 (0.03)	0.23 (0.03)
BM42	A_a+x_	2.47 (0.23)	0.35 (0.11)	1.71 (0.09)	2.82 (0.11)	0.68 (0.04)	0.35 (0.02)
	G_a+x_	3.09 (0.29)	0.30 (0.10)	1.70 (0.09)	2.82 (0.11)	0.68 (0.04)	0.40 (0.03)
	H_a+x_	3.28 (0.31)	0.34 (0.11)	1.70 (0.09)	2.82 (0.11)	0.68 (0.04)	0.41 (0.03)
BM63	A_a+x_	3.89 (0.33)	0.77 (0.20)	1.19 (0.10)	4.73 (0.17)	0.80 (0.05)	0.41 (0.02)
	G_a+x_	4.89 (0.42)	0.64 (0.16)	1.19 (0.10)	4.73 (0.17)	0.80 (0.05)	0.45 (0.02)
	H_a+x_	5.17 (0.44)	0.72 (0.19)	1.19 (0.10)	4.73 (0.17)	0.80 (0.05)	0.47 (0.02)
BMM	A_a_	5.59 (0.46)		1.24 (0.16)		5.74 (0.24)	0.45 (0.03)
	G_a_	7.01 (0.57)		1.23 (0.16)		5.75 (0.24)	0.50 (0.03)
	H_a_	7.42 (0.61)		1.23 (0.16)		5.75 (0.24)	0.52 (0.03)

Traits: litter size (LS0), litter weight (LW0), body weights at ages of 21, 42, 63 days and at mating (BM21, BM42, BM63, BMM). Kinds of relationship matrices: pedigree-derived numerator relationship matrix (A), gene-drop derived (G), combined expected and observed genomic relationships (H); subscripts indicate that autosomal relationships only (a) or both autosomal and X-chromosomal relationships (a+x) were part of the model. Genetic parameters: $\sigma_{a}^{2}$: autosomal additive genetic variance, $\sigma_{x}^{2}$: X-chromosomal additive genetic variance, $\sigma_{c}^{2}$: common litter environmental variance, $\sigma_{p}^{2}$: permanent environmental variance, $\sigma_{e}^{2}$: residual variance, $h^{2}$: heritability, standard errors in brackets.

The proportion of the total genetic variance that was attributed to the sex chromosome was approximately 3% for BM21 in all analyses (data in Table 6). When founders were assumed to be related, the same proportion was 9% and 12% for BM42 and BM63, respectively. These were lower than the comparative values of 12% and 17%, when the assumption of unrelatedness was applied. Over all traits, a standard pattern emerged (Table 6) were genetic variance components and heritability were larger when either an H or a G matrix was part of the model, compared to an A matrix analysis. In contrast, results from H and G matrices were almost equal for all traits. Estimated residual and common litter environmental variances were barely affected by the choice of genetic relationship matrix for any of the traits analyzed. The same was true for the permanent environmental variance component for BM42 and BM63, whereas both litter traits displayed lower estimates for the permanent environmental variance component from H and G matrices, compared with A matrices. This was accompanied by considerably larger estimates for autosomal additive genetic variances. Consequently, heritability for LS0 was 20% when founder relationships were taken into account (matrix **H**), vs. 12% when they were not taken into account (Table 6). For LW0 and growth traits, the respective comparisons were 34% and 32% vs. 23% (LW0), 23% and 22% vs. 17% (BM21), 41% and 40% vs. 35% (BM42), 47% and 45% vs. 41% (BM63), and 52% and 50% vs. 45% (BMM). For LS0, in particular, the increase in the estimates of the genetic variance using **H** and **G** matrices is larger than 60% (64% and 78%). To that effect estimated genetic standard deviations changed and the corresponding range of genotypes with very low and very high litter size, defined as six times the estimated genetic standard deviation, rose from seven to approximately nine pups per litter. In essence, the results from Table 6 demonstrate that the chosen scaling of H and G matrices provided higher estimates for the genetic variance components for all traits. This increase can be interpreted as caused by correctly including the segregation variance, which is part of the genetic variance from generation F_2_ onwards and is expected to be prominent for the trait LS0, on which the FL1 line has been selected.

Our own estimated heritability values for LS0 are within the wide range of results reported in older studies. Falconer (1960) reported 8.3% heritability for upward selection and 22.9% for downward selection. Bradford (1968) presented realized heritability from 0.13 to 0.39 for litter size in several lines, while Bakker *et al.* (1978) found a realized heritability of 0.11. More recent investigations tend toward lower values, compared with our result of 19%, *e.g.*, Beniwal *et al.* (1992) reported a comparatively low heritability for litter size (0.181 ± 0.093 for control and 0.166 ± 0.043 overall), as did Peripato *et al.* (2004), with *h*^2^ = 12%. Similarly, Gutiérrez *et al.* (2006) published heritability values for litter size from 0.099 to 0.101. Gutiérrez *et al.* (2006) also reported considerably lower heritability, from 0.112 to 0.148 (derived with different models), for litter weight.

Estimated heritability values for body weight traits in mice vary widely in the literature. Falconer (1953) reported the heritability for body weight measured at day 60 as approximately 20% for upward selection and 50% for downward selection. Interestingly, Wilson *et al.* (1971) found that the realized heritability for body weight at day 60 declined from 0.32 in the first 10 generations to 0.08 between generation 61 and 70, in a selection experiment. Eisen (1978) reported a heritability of 0.44 (or 0.55, depending on the method of estimation) for six week body weight. Heath *et al.* (1995) reported a heritability of 0.25 before selection and a mean heritability of 0.216 ± 0.0077 (from 0191 ± 0.016 to 0.242 ± 0.014 for different pairs of lines) for six week body weight. In an intercross-population, Kramer *et al.* (1998) estimated high heritability values of 0.54 ± 0.24 for three week body weight, 0.76 ± 0.04 for six week body weight and 0.81 ± 0.01 for nine week body weight in a cross-fostering experiment. Although they are within the upper ranks, our estimates of approximately 40–50% at ages from 42 to 63 days may be seen as well within the range of literature values.

#### Estimated genetic trends:

**Figure 3:**
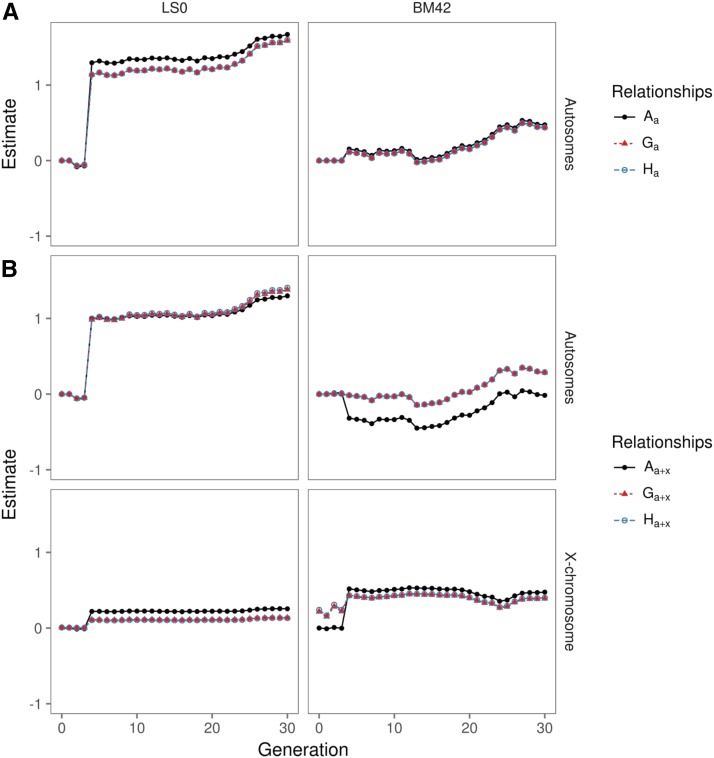
Autosomal (above and middle) and X-chromosomal (below) genetic trends for litter size (LS0, left) and body mass at day 42 (BM42, right) obtained by different kinds* of relationship matrices (A, G and H) and models with (subscript a+x) and without (subscript a) taking X-chromosomal genetic variation into account. *) Kinds of relationship matrices: pedigree-derived numerator relationship matrix (A), gene-drop derived (G), combined expected and observed genomic relationships (H); subscripts indicate that autosomal relationships only (a) or both autosomal and X-chromosomal relationships (a+x) were fitted.

Figure 3 shows estimated genetic trends for LS0 and BM42 from models with different relationship matrices. The upper panels (Figure 3A) show genetic trends from models with autosomal relationships only, while the other panels (Figure 3B) depict those from applying the autosomal and X-chromosomal relationship matrices, simultaneously. In general, trends are very similar by shape and also by level, in accordance with the absence of any serious trend. An exception is the jump at generation F_3_ for LS0. This reflects the fact that from generation F_3_ onwards only the descendants of a single family were maintained in order to breed the later generations, which makes the differences between the four founder families manifest. See also Figure S2 for genetic trends in other traits.

#### Underlying assumptions:

In deriving Ã, the expected overall heterozygosity, as described by the scale parameter S, is taken as being fully determined by the observed allele frequencies of the genotyped founders and the assumption that both lines contribute equally to the new composite population. The latter can easily be adapted to more than two founder lines, even with unequal contributions, by an alteration of the definition of the average allele frequency $\bar{p}$. With more than two founder lines, the equilibrium state with foreseen autosomal heterozygosity, $S=\sum {\bar{p}}_{i}\left(1-{\bar{p}}_{i}\right)$, may also be reached later than in the second crossbred generation. This depends on the mating scheme that generates the new composite population and, as with two founder lines, is an asymptotic process for X-chromosome markers. The initial numbers of male and female founders may be different in each line to be crossed later. Autosomal and X-chromosome reference populations should be comparable, especially in the interpretation of genetic parameters. Therefore, it seems reasonable to define $\bar{p}$ for X-chromosome markers, and hence S, as if males and females from all founder lines contribute equally. However, this does not need to be the case, as demonstrated by our mouse example. A further assumption entered into Ã is that founder genotype frequencies meet line-specific Hardy-Weinberg equilibrium. Observed founder genomic self-relationships that deviate from their expected counterparts indicate an excess or lack of heterozygosity relative to this assumption. Actual marker heterozygosity values are then accounted for using the combined relationship matrix H.

Matrix Ã reflects average genomic relationships that result from repeatedly sampling alleles at observed line-specific frequencies from founders and their forward transmission to later generations, in accordance with the pedigree. No attempts were made to estimate IBD-based founder relationships (*e.g.*, Powell *et al.* 2010) within lines. There are typically only a few founders of experimental populations, as in our mouse example, and they may provide information on within-line IBD-relationships only with high sampling errors, unless a larger sample is genotyped. However, we did not treat founders as a sample from their respective lines but their genotypes were treated as a complete inventory of all possible alleles that can be further transmitted to the F_2_ generation and beyond. Therefore, they fully determine the genetic makeup of the later crossbred population, as mirrored by the construction of Ã. The usefulness of Ã, and hence H, for estimating the genetic variance will depend on how well the frequency spectrum of markers reflect the frequency spectrum of QTL (quantitative trait loci) for a trait (Walsh and Lynch 2018, p. 631-668). The latter requirement is likely to be largely fulfilled in a cross of two divergently selected lines, where a large contribution by segregation variance can be expected to be picked up by a large proportion of markers with line-specific alleles.

#### Fields of application:

In the analysis of selection experiments, mixed models have largely replaced other methods due to their flexibility (Walsh and Lynch 2018, p. 631-668). Data from AIL lines can be seen as a special case, with no artificial selection applied. Pedigree-based relationship matrices traditionally treat founders as unrelated and non-inbred. As only a limited number of founders are often genotyped, due to cost, this unrealistic assumption can be overcome by taking genomic founder relationships into account. Using mixed models, with the described Ã based version of H, accounts for the initial disequilibrium in allele and genotype frequencies, which exists for more generations on the X chromosome, compared with only two generations for autosomal loci. Meaningful estimates of genetic variances and heritability may be calculated in crosses of two lines in which line-specific alleles can be expected to prevail both at marker loci and QTL due to divergent selection histories. Moreover, in contrast to a gene-drop derived matrix, additional genotypes from later generations can easily be integrated by setting up a joint genomic relationship matrix for all genotyped individuals and joining it with Ã into H. Thus Ã leads to more realistic assumptions and more flexibility in the analysis of selection experiments if all founders are genotyped.

### Conclusion

An approach for constructing expected autosomal and X-chromosomal genomic relationship matrices for founders from an arbitrary number of founder lines was developed. Extension to non-genotyped individuals in later generations can be performed by an adapted version of the tabular method using pedigree information. The resulting matrix Ã expresses relationships as average genomic relationships, as one would expect from repeated random sampling of alleles from founders at the observed frequencies. Implicitly, Ã accounts for any proportion of segregation variance between zero and one, which is not possible using only pedigree data. Observed marker data of founders and non-founders can then be combined into a joint relationship matrix, H, and its inverse can be used in mixed models for estimating the genetic variance in the crossbred population.
