# Corrigendum

**DOI:** 10.1534/g3.119.400288

**Published:** 2019-05-27

In the article by J. Meng, M. Mayer, E. Wytrwat, M. Langhammer, and N. Reinsch (*G3: Genes|Genomes|Genetics* 9(3): 889-899), entitled “Turning Observed Founder Alleles into Expected Relationships in an Intercross Population,” on page 894, the authors have updated the Data Availability Statement to include a link to the RADAR (research data repository) repository, https://doi.org/10.22000/155, where they have uploaded two example programs, one for autosomal and the other for X-chromosomal relationships, written in R and accompanied by example input and output files, as well as a short description.

